# Rad6B acts downstream of Wnt signaling to stabilize β-catenin: Implications for a novel Wnt/β-catenin target

**DOI:** 10.1186/1750-2187-6-6

**Published:** 2011-07-18

**Authors:** Brigitte Gerard, Larry Tait, Pratima Nangia-Makker, Malathy PV Shekhar

**Affiliations:** 1 Department of Oncology, Wayne State University, 110 East Warren Avenue, Detroit, 48201, Michigan; 2 Department of Pathology, Wayne State University, 110 East Warren Avenue, Detroit, 48201, Michigan; 3 Karmanos Cancer Institute, 110 East Warren Avenue, Detroit, 48201, Michigan

## Abstract

**Background:**

Aberrant Wnt/β-catenin signaling is associated with breast cancer even though genetic mutations in Wnt signaling components are rare. We have previously demonstrated that Rad6B, an ubiquitin conjugating enzyme, stabilizes β-catenin via polyubiqutin modifications that render β-catenin insensitive to proteasomal degradation. Rad6B is a transcriptional target of β-catenin, creating a positive feedback loop between Rad6B expression and β-catenin activation.

**Methods:**

To isolate subpopulations expressing high or low Rad6B levels, we transfected MDA-MB-231 or WS-15 human breast cancer cells with ZsGreen fluorescent reporter vector in which the expression of ZsGreen was placed under the control of Rad6B promoter. ZsGreen^high ^and ZsGreen^low ^subpopulations, reflective of high and low Rad6B promoter activity, respectively, were isolated by FACS. To determine the relevance of Wnt signaling in Rad6B-mediated β-catenin stabilization/activation, the ZsGreen^high ^cells were transfected with signaling-defective Wnt coreceptor LRP6Δ173. Rad6B expression and promoter activity were determined by RT-PCR, Western blot and Rad6B promoter-mediated luciferase assays. β-catenin levels and transcriptional activity were determined by Western blot and TOP/FOP Flash reporter assays. Tumor formation and morphologies of ZsGreen^low^, ZsGreen^high^, and ZsGreen^high^/LRP6Δ173 cells compared to unsorted vector controls were evaluated in nude mice. Expression of Wnt signaling related genes was profiled using the Wnt signaling pathway RT2 Profiler PCR arrays.

**Results:**

ZsGreen^high ^subpopulations showed high Rad6B expression and Rad6B promoter activity as compared to ZsGreen^low ^cells. ZsGreen^high ^(high Rad6B expressors) also showed elevated β-catenin levels and TOP/Flash activity. Inhibiting Wnt signaling in the high Rad6B expressors decreased ZsGreen fluorescence, Rad6B gene expression, β-catenin levels and TOP/Flash activity. Tumors derived from high Rad6B expressors were predominantly composed of cells with epithelial mesenchymal transition (EMT) phenotype as compared to control tumors that were composed of both cuboidal and EMT-type cells. Tumors derived from low Rad6B expressors lacked EMT phenotype. Inhibition of LRP6 function in the high Rad6B expressors abrogated the EMT phenotype. Gene expression profiling showed upregulation of several Wnt signaling pathway regulators in high Rad6B expressors that were downregulated by interference of Wnt signaling with mutant LRP6 or by Rad6B silencing.

**Conclusions:**

These data reveal a functional link between the canonical Wnt pathway and Rad6B in β-catenin activation and breast cancer progression.

## Background

The canonical Wnt signaling pathway regulates several processes including early neoplasia. Activation of the canonical Wnt pathway involves stabilization of β-catenin through the binding of Wnt ligands to the cell surface Frizzled (Fz) family receptors and low density lipoprotein receptor (LDLR)-related protein 5 (LRP5) and LRP6. The major output of this pathway is nuclear translocation of β-catenin, which stimulates expression of β-catenin responsive target genes that promote cell proliferation, differentiation, and invasion [1-4]. In the absence of Wnt ligands, β-catenin is phosphorylated by a multiprotein degradation complex involving APC, Axin, GSK3β and casein kinase 1, which marks it for ubiquitination and degradation by the 26S proteasome [5,6]. Although genetic mutations of APC, Axin or β-catenin are involved in the development of several types of cancer, they are rarely observed in breast cancer [7]. However, compelling evidence has indicated that abnormal regulation of Wnt/β-catenin signaling can lead to mammary carcinogenesis [8-13]. Upregulated nuclear/cytoplasmic β-catenin is found in 40-60% of human breast cancer specimens and correlates with poor prognosis [14,15]. This suggests that alternate/additional mechanisms of β-catenin stabilization may be the underlying cause(s) of aberrant β-catenin activation in breast cancer patients. Overexpression of Wnt ligands Wnt 1, Wnt 10 b or an activated form of β-catenin in mice results in mammary tumors [16,17]. In human breast cancer, secreted Frizzled protein (sFRP-1), a member of the Wnt antagonist family, is downregulated in malignant tissues [18,19]. Expression of Wnt coreceptors LRP6, but not LRP5, was found to be upregulated in a subset of human breast carcinomas, and downregulation of LRP6 was sufficient to inhibit breast carcinogenesis [20]. We have previously demonstrated that Rad6B, a 17-kDa ubiquitin conjugating enzyme [21], stabilizes β-catenin by inducing K63-linked β-catenin polyubiquitination, which renders β-catenin insensitive to 26S proteasomal degradation [22]. Rad6B silencing decreases polyubiquitinated β-catenin levels and activity, and suppresses the epithelial mesenchymal transition (EMT) phenotype of WS-15 human breast cancer cells [22]. Rad6B expression is low in normal human breast tissues, but increases in Rad6B expression is observed in early breast cancer with frequent overexpression in breast carcinomas [23]. Rad6B itself is a transcriptional target of ∃-catenin/T-Cell Factor (24), suggesting the presence of a positive feedback loop between Rad6B gene expression and β-catenin stabilization.

Here we determined if Rad6B mediated β-catenin stabilization in breast cancer cells requires intact Wnt signaling. Using the human Rad6B promoter to direct expression of ZsGreen reporter protein, we isolated breast cancer subpopulations expressing high and low levels of Rad6B, and demonstrated β-catenin activation in high Rad6B subpopulations. Further, the Rad6B-mediated β-catenin activation requires intact Wnt signaling since disruption of Wnt signaling in high Rad6B expressors with a signaling defective LRP6, decreases β-catenin levels and activity, and Rad6B promoter-directed reporter and Rad6B gene expression. Breast cancer subpopulations selected for high Rad6B produce tumors with the EMT phenotype, which is suppressed by blocking LRP6 function. These data suggest that Rad6B functions downstream of Wnt signaling in β-catenin stabilization and breast cancer progression.

## Methods

### Cell culture

MDA-MB-231 (American Type Culture Collection), WS-15 (Cell Core Facility of Karmanos Cancer Institute), pLKO-Rad6BshRNA and empty pLKO WS-15 human breast cancer [22] cells were maintained in Dulbecco's modified Eagle/F-12 medium supplemented with 5% fetal bovine serum.

### Plasmids

The human Rad6B (R6B) promoter sequence (bases -401/+9 relative to the ATG start codon +1; GenBank:NM_003337[24]) was subcloned into the XhoI/HindIII sites of promoterless pZsGreen1 reporter vector. The integrity of the cloned sequence was verified by DNA sequencing. Construction and activity of the human Rad6B promoter subcloned into pGL3-Basic reporter vector has been described previously [24]. LRP6Δ173 lacking the C-terminal 173 amino acids subcloned into pcDNA3.1 and tagged at the C-terminus with myc and polyhistidine tags was a generous gift from Dr. Anthony Brown at Weill Medical College of Cornell University (25). LRP6Δ173 lacks the PPP(S/T)Px(S/T) motifs that are critical for efficient signaling [25,26].

### Transfections and Fluorescence-activated cell sorting

MDA-MB-231 cells were transfected with pR6B promoter-ZsGreen1 or the corresponding promoterless vector with Metafectene (Biontex) and clones selected with G418. Single cell suspensions of pooled clones from R6B promoter-ZsGreen transfections were sorted in BD FACSDiVa, and the top and bottom 10% of cells with the highest (referred as R6B-Zs^high^) and lowest (referred as R6B-Zs^low^) ZsGreen fluorescence, respectively, were collected into 50% FBS in Phosphate buffered saline and propagated. R6B-Zs^high ^cells were stably transfected with pcDNA-LRP6Δ173 or empty vector, and ZsGreen fluorescence analyzed by flow cytometry.

### Immunohistochemistry, immunofluorescence, and Western blotting

Immunohistochemistry and immunofluorescence analyses were performed on paraffin-embedded xenografts or cells as previously described [22]. For immunohistochemical analysis, proteins were detected with appropriate biotinylated secondary antibodies and HRP-conjugated streptavidin. Nuclei were counterstained with hematoxylin. For immunofluorescence analysis, proteins were detected with FITC- or Texas Red-conjugated secondary antibodies, and counterstained with DAPI. Slides were stained in the absence of primary antibody or with isotype-matched nonimmune IgG to assess nonspecific reactions. Images were collected on Olympus BX60 microscope equipped with Sony high resolution/sensitivity CCD video camera. The antibodies used for immunohistochemistry and/or immunofluorescence were: anti-Rad6 [22,23], anti-β-catenin (SantaCruz [22]), anti-myc tag (gift from Dr. Guri Tzivion, University of Mississippi), anti-Vimentin (Abcam), and anti-Snail1 (Abcam). Since our Rad6 antibody does not distinguish the two isoforms Rad6A and Rad6B, we have indicated the detected protein as Rad6. Mass spectrometry analysis of proteins immunoprecipitated from human breast cells using our anti-Rad6 antibody identified only Rad6B peptides, indicating that Rad6B is the major isoform in breast cells (unpublished data). Moreover, the expression levels of Rad6 protein correlate with the molecular data (Rad6B promoter assays and Rad6B RNAi), confirming the identity of Rad6B detected by our antibody. Cytosols were prepared from control, R6B-Zs^high^, R6B-Zs^low^, and R6B-Zs^high^/LRP6Δ173 MDA-MB-231 cells, and control, R6B-Zs^high ^and Rad6BshRNA WS-15 [22] cells as previously described, and analyzed by immunoblotting for Rad6, β-catenin, LRP6, myc-tagged mutant LRP6 or β-actin.

### Luciferase assays and gene expression analysis

To assay the transcriptional activity of ∃-catenin, cells were transiently transfected with a mixture (40:1) of inducible (pTOP/Flash) or mutant (pFOP/Flash) TCF-responsive firefly luciferase (Upstate Biotechnology) and pRLTK (Promega) vectors as previously described [22]. The R6B promoter-luciferase reporter vector [24] was substituted for pTOP/Flash in some assays to assess Rad6B promoter activity. All experiments were performed thrice in duplicate. Firefly and Renilla activities in lysates were assayed with a Dual Luciferase Reporter Assay System (Promega).

### Semi-quantitative RT-PCR

Total RNA was prepared from control, R6B-Zs^high ^and R6B-Zs^high^/LRP6Δ173 MDA-MB-231 cells with TRIzol reagent (Invitrogen). Total RNA (1.0 μg) was reverse-transcribed using Superscript III (Invitrogen), and Rad6B cDNA was PCR amplified with +17/+33 and +114/+97 [GenBank:NM_003337] forward and reverse primers, respectively [22]. GAPDH expression was monitored by amplifications with forward (+186/+206) and reverse (+320/+302) [GenBank:XM_006959] primers. The reaction conditions that yielded a detectable product with the minimum number of cycles were employed: 95°C, 1 min/55°C, 1 min/65°C, 2 min for 21 cycles.

### Real time RT-PCR analysis of Wnt signaling genes

The human Wnt signaling pathway RT^2 ^Profiler PCR arrays (SuperArray Bioscience) were used to profile the expression of 84 genes related to Wnt signaling. Total RNA was extracted from R6B-Zs^high^, R6B-Zs^high^/LRP6Δ173, Rad6BshRNA MDA-MB-231 or WS-15 cells and their controls with TRIzol. Single stranded cDNA was synthesized from 2 μg of total RNA by using the SuperArray reaction ready first strand cDNA synthesis kit. The cDNAs were mixed with SuperArray RT^2 ^Real time SYBR Green/ROX PCR master mix and real time PCR performed in accordance with the manufacturer's instructions. Thermal cycling and fluorescence detection were performed using an ABI Prism 7700 Sequence Detection System (Applied Biosystems), and expression of Wnt regulated transcripts were compared between the groups.

### In vivo assays

Xenografts of MDA-MB-231 or WS-15 derived subpopulations were generated by injecting 1 × 10^6 ^or 5 × 10^6 ^MDA-MB-231 or WS-15 derivatives, respectively, in 0.1 ml serum-free media or Matrigel subcutaneously near the nipple of gland #5 of female nude mice. Xenografts were removed at 50 days and fixed in buffered-formalin. *In vivo *experiments were approved by the Institutional Animal Care and Use Committee, and conformed to the NIH regulatory standards.

### Terminal deoxynucleotidyl transferase biotin-dUTP nick end-labeling (TUNEL)

TUNEL staining was performed as previously described [27]. Apoptotic cells in MDA-MB-231 xenografts were identified on deparaffinized sections using the Deadend fluorimetric TUNEL system (Promega). Sections were counterstained with propidium iodide. Images were captured with an Olympus BX40 microscope and processed with the M5+ microcomputer imaging device (Interfocus Imaging, Ltd., Cambridge, U.K.).

### Statistical Analysis

Data were analyzed with GraphPad software using either Student's t test or ANOVA. P < 0.05 was considered significant.

## Results

### Tumor subpopulations with endogenous Rad6B overexpression show elevated β-catenin levels and activity

**Figure 1:**
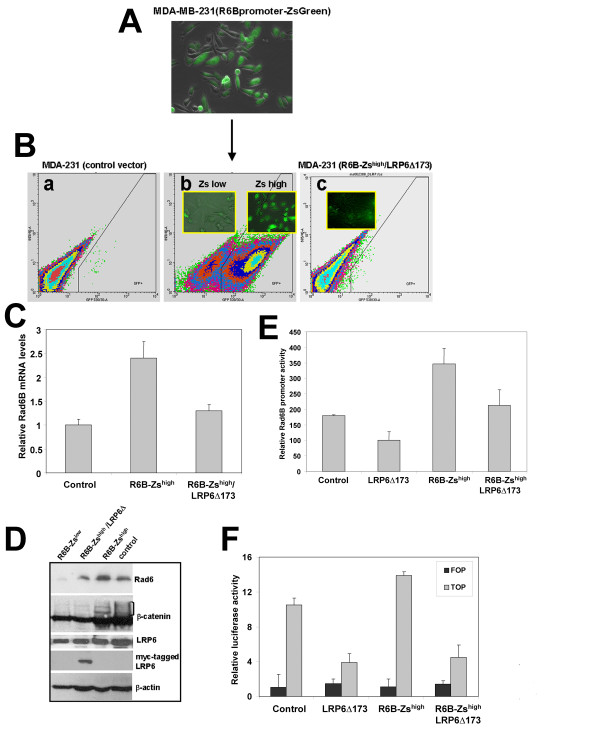
**Breast cancer subpopulations selected for high Rad6B promoter activity display elevated β-catenin levels and activity, which is suppressed by LRP6 inhibition**. (A) Fluorescence/phase contrast images of MDA-MB-231 cells stably transfected with Rad6B promoter-ZsGreen1 reporter vector. (B) FACS analysis of MDA-MB-231 cells transfected with promoterless (a) or Rad6B promoter-ZsGreen1 (b) vector. Panel c, FACS profile of R6B-Zs^high ^cells transfected with mutant LRP6Δ173. Insets in b represent fluorescence images of sorted subpopulations with low (Zs low) and high (Zs high) green fluorescence. (C) Rad6B mRNA expression relative to GAPDH in the indicated cells determined by semi-quantitative RT-PCR. (D) Steady-state levels of indicated proteins in the cytosols of indicated MDA-MB-231 subpopulations. (E) Relative Rad6B promoter activity in MDA-MB-231 cells transfected with Rad6B promoter-luciferase and pRLTK. (F) Relative pTOP/Flash or pFOP/Flash reporter activities in MDA-MB-231 cells. The data shown in E and F are averages (± S.E.M) of three separate experiments performed in duplicate.

We have previously demonstrated a positive association between Rad6B and β-catenin expression in breast carcinomas [24]. To establish the functional link between Rad6B expression and β-catenin activity, MDA-MB-231 cells were stably transfected with either the pZsGreen1 reporter vector in which the expression of ZsGreen1 reporter is placed under the control of the human Rad6B promoter (Figure 1A) or the control promoterless vector. MDA-MB-231 cells were chosen because they have autocrine Wnt signaling activity [28]. MDA-MB-231 subpopulations with high (R6B-Zs^high^) and low (R6B-Zs^low^) promoter activities were isolated by cell sorting, and expanded for further analysis (Figure 1Bb). As shown in Figure 1B, strong expression of ZsGreen1 reporter is reflective of strong Rad6B promoter activity (compare panels a and b in Figure 1B). To determine the relationship between Rad6B and Wnt signaling in β-catenin stabilization, R6B-Zs^high ^cells were stably transfected with LRP6Δ173, a signaling defective dominant negative mutant of LRP6 Wnt coreceptor [25]. This mutant LRP6 lacks the cytoplasmic tail (c-tail) containing the stability elements that are required for Wnt1- and Dvl-dependent signaling [25,26]. Ectopic expression of LRP6Δ173 diminished ZsGreen1 fluorescence in R6B-Zs^high ^cells (compare panels b and c in Figure 1B) suggesting that disruption of Wnt signaling decreases Rad6B promoter activity. RT-PCR analysis showed ~2.5 fold increase in Rad6B mRNA levels in R6B-Zs^high ^cells as compared to control MDA-MB-231 cells, and ectopic mutant LRP6Δ173 decreased Rad6B gene expression in R6B-Zs^high ^cells (P < 0.01; Figure 1C). R6B-Zs^high ^cells expressed higher steady state levels of Rad6 protein as compared to control or R6B-Zs^low ^cells (Figure 1D), and expression of mutant LRP6 (as confirmed by myc tag antibody) in R6B-Zs^high ^cells decreased Rad6 protein levels (Figure 1D). Simultaneous measurement of β-catenin protein levels showed elevated levels of high molecular weight β-catenin fraction in control and R6B-Zs^high ^cells as compared to R6B-Zs^low ^cells, and ectopic LRP6Δ173 expression in R6B-Zs^high ^cells dramatically reduced the high molecular weight β-catenin fraction (Figure 1D). The steady-state levels of endogenous LRP6 (detected with an antibody reactive to its C-terminus) in the MDA-MB-231 subpopulations did not show obvious relationship with Rad6 levels. Rad6B promoter activity measurements corroborated ZsGreen fluorescence and Rad6B gene expression data. R6B-Zs^high ^MDA-MB-231 cells exhibited higher Rad6B promoter-driven luciferase expression as compared to control, and introduction of LRP6Δ173 into R6B-Zs^high ^cells decreased luciferase expression (P < 0.05; Figure 1E). The transcriptional activity of endogenous β-catenin was measured by TOP/FOP Flash reporter assays. High levels of β-catenin-mediated TOP/Flash activity observed in R6B-Zs^high ^cells were significantly downregulated by LRP6Δ173 (P < 0.01; Figure 1F). These data suggest that interference of Wnt signaling in Rad6B-overexpressing subpopulations downmodulates intracellular β-catenin and Rad6B gene expression, confirming the positive feedback relationship between Rad6B and β-catenin levels previously reported [22,24].

**Figure 2:**
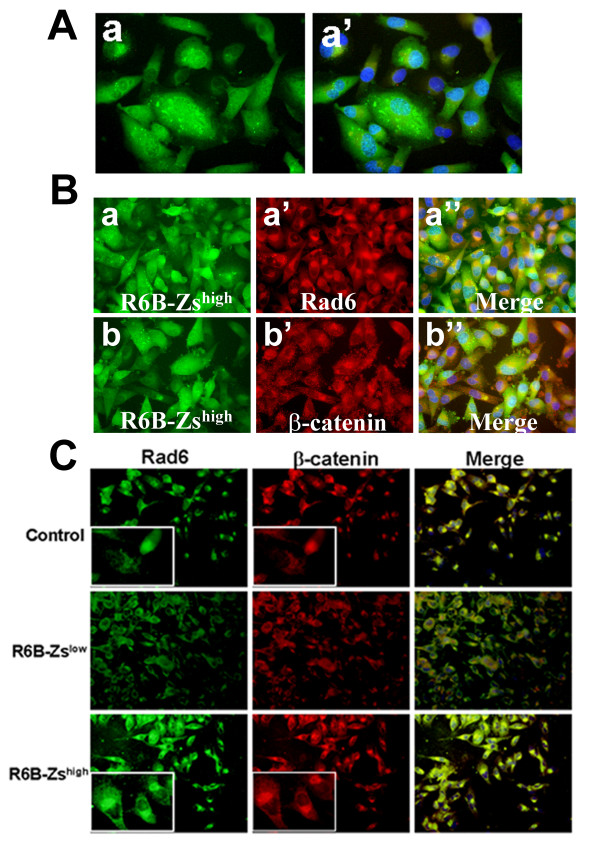
**Subpopulations selected for high endogenous Rad6B expression (ZsGreen^high^) show elevated intracellular β-catenin**. (A) ZsGreen fluorescence imaging of R6B-Zs^high ^MDA-MB-231 cells. a' is ZsGreen fluorescence merged with DAPI. Magnification ×20. (B) Immunofluorescence staining of R6B-Zs^high ^MDA-MB-231 cells with anti-Rad6 or anti-β-catenin, and detected with appropriate Texas Red conjugated secondary antibody. Endogenous ZsGreen fluorescence was merged with Texas Red and DAPI. Original magnification, ×40. (C) Immunofluorescence staining of control, R6B-Zs^high ^and R6B-Zs^low ^MDA-MB-231 cells with anti-Rad6 or anti-β-catenin antibodies. Proteins were detected with FITC (Rad6) or Texas Red (β-catenin) conjugated secondary antibodies. Original magnification, ×20.

**Figure 3:**
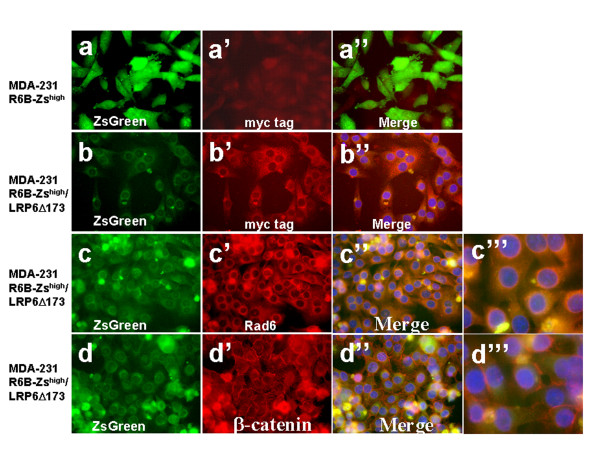
**Wnt disruption in high Rad6B expressors decreases intracellular β-catenin, Rad6B promoter-directed ZsGreen expression, and suppresses EMT phenotype**. Immunofluorescence staining of control R6B-Zs^high ^or R6B-Zs^high^/LRP6Δ173 MDA-MB-231 cells with anti-myc tag (a', b'), anti-Rad6 (c'-c''') or anti-β-catenin (d'-d''') antibodies. Proteins were detected with Texas Red conjugated secondary antibodies. Endogenous ZsGreen fluorescence was merged with Texas Red and DAPI. Original magnification, ×20 for all panels except c''' and d''', X100.

We have previously demonstrated that silencing Rad6B gene expression decreases intracellular β-catenin without affecting the cell membrane associated β-catenin and restores epithelial polarity [22]. To determine whether this relationship between Rad6B and β-catenin is dependent upon Wnt signaling, the R6B-Zs^high ^MDA-MB-231 cells (Figure 2A) were analyzed for expression/distribution of Rad6 and β-catenin by immunofluorescence staining. As expected, staining with anti-Rad6 antibody corroborated the strong association between Rad6B promoter-driven ZsGreen fluorescence and Rad6 protein detected with Texas Red conjugated secondary antibody (Figure 2Ba-a''). R6B-Zs^high ^cells also displayed strong β-catenin staining in the cytoplasm (Figure 2Bb-b''). The association between Rad6B and β-catenin accumulation was also verified by immunofluorescence analysis of Rad6 and β-catenin in vector control, R6B-Zs^high ^and R6B-Zs^low ^MDA-MB-231 cells. Strong Rad6 and β-catenin staining that colocalized at the periphery of nucleus was observed in majority of the R6B-Zs^high ^cells as compared to control cells, whereas R6B-Zs^low ^cells displayed weak and diffuse reactivities to Rad6 and β-catenin antibodies (Figure 2C). To determine the effect of inhibiting Wnt signaling on expression/distribution of Rad6 and β-catenin, immunofluorescence staining was performed on control R6B-Zs^high ^(Figure 3a) or R6B-Zs^high^/LRP6Δ173 (Figure 3b-d) MDA-MB-231 cells. LRP6Δ173 expression was verified by immunostaining with anti-myc tag antibody (Figure 3b'). Mutant LRP6 induced a dramatic drop in ZsGreen fluorescence (compare Figure 3b, c and 3d with Figure 3a) and concomitant Rad6 protein staining (compare Figure 3c'-c''' with Figure 2Ba'). Unlike in R6B-Zs^high ^cells where strong β-catenin staining was observed in the cytoplasm (Figure 2Bb'), in R6B-Zs^high^/LRP6Δ173 cells cytoplasmic β-catenin staining was diminished and β-catenin was predominantly confined to the cell membranes (Figure 3d'-d'''). Downregulation of intracellular β-catenin and restoration of β-catenin to the cell membranes was accompanied by conversion from spindly (mesenchymal) to cuboidal (epithelial) morphology (compare Figure 3a with Figure 3b, c and 3d). These alterations in β-catenin staining patterns and cell morphologies induced by Wnt disruption resemble those induced by Rad6B silencing [22].

### Rad6B overexpressing breast cancer subpopulations produce tumors with homogeneous epithelial mesenchymal transition (EMT) phenotype

**Figure 4:**
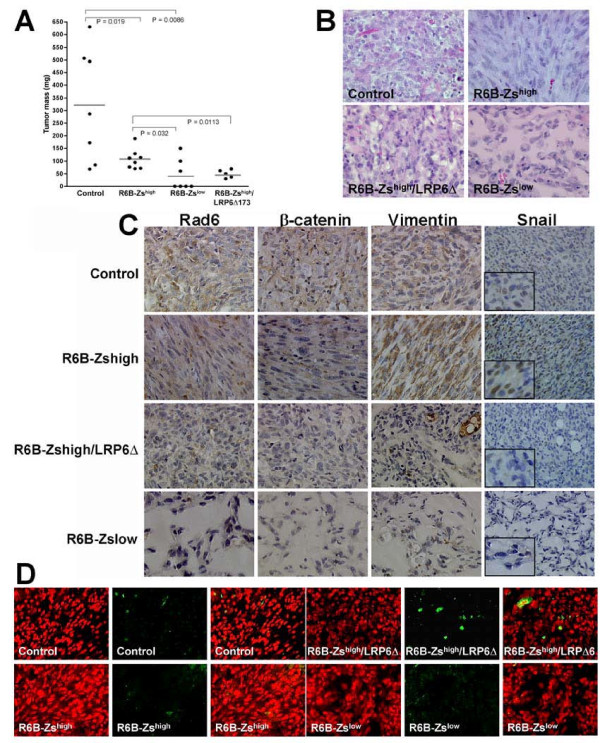
**Tumors derived from high Rad6B expressors show homogeneous EMT phenotype that is suppressed by mutant LRP6**. (A) Tumor masses produced by the indicated MDA-MB-231 subpopulations. (B) Tumor morphologies of xenografts derived from the indicated MDA-MB-231 subpopulations by H&E staining. (C) Immunohistochemical analysis of Rad6, β-catenin, Vimentin and Snail1 in vector control, R6B-Zs^high^, R6B-Zs^high^/LRP6Δ173 and R6B-Zs^low ^tumors. Original magnification ×20. (D) TUNEL staining (green) of tumors produced by the indicated MDA-MB-231 subpopulations. Sections were counterstained with propidium iodide. Original magnification ×40.

To analyze the functional contribution of Rad6B to breast cancer development, vector control, R6B-Zs^high^, or R6B-Zs^low ^MDA-MB-231 cells were implanted s.c. into the mammary fatpads of female nude mice. Vector control MDA-MB-231 cells produced tumors that displayed heterogeneity in tumor mass (Figure 4A). Tumors arising from R6B-Zs^high ^cells were significantly smaller than controls (*P *= 0.02), but were significantly larger (*P *= 0.03) than those produced by R6B-Zs^low ^cells. To determine the contribution of Wnt signaling in Rad6B mediated breast cancer progression, R6B-Zs^high^/LRP6Δ173 cells were implanted into the mammary fatpads of female nude mice. Inhibition of LRP6 function in Rad6B-overexpressing subpopulations resulted in a significant decrease of tumor growth (*P *= 0.01) as compared to R6B-Zs^high ^cells (Figure 4A). Histologic analysis revealed remarkable differences in the tumor morphologies. Tumors derived from R6B-Zs^high ^cells were mainly composed of spindle shaped cells in contrast to vector controls that consisted of both cuboidal and spindle shaped cells (Figure 4B). Consistent with their smaller size, tumors derived from R6B-Zs^low ^cells were sparsely populated and lacked spindle shaped cells (Figure 4B). In agreement with the *in vitro *data of Figure 3, Wnt signaling-disrupted R6B-Zs^high ^cells produced tumors that lacked the EMT phenotype of R6B-Zs^high ^cells (Figure 4B). Consistent with their EMT phenotype, strong expression of EMT markers Vimentin and Snail1 was observed in R6B-Zs^high ^tumors as compared to control tumors that expressed variable levels of these markers (Figure 4C). Expression of Rad6, β-catenin, Vimentin and Snail1 were all downregulated in R6B-Zs^high^/LRP6Δ173 tumors, and were negligible in R6B-Zs^low ^tumors (Figure 4C). These data suggest that Rad6B overexpression positively contributes to tumor growth and to EMT, and these Rad6B-mediated events are dependent upon β-catenin activation and intact Wnt signaling. TUNEL positive cells were detected in R6B-Zs^high^/LRP6Δ173 MDA-MB-231 derived tumors suggesting that Rad6B expression and intact Wnt/β-catenin signaling are required for tumor maintenance. Only rare TUNEL positive cells were observed in R6B-Zs^low ^tumors suggesting that their reduced tumor growth is not due to enhanced apoptosis (Figure 4D).

### General relevance of Rad6B and Wnt/β-catenin link

**Figure 5:**
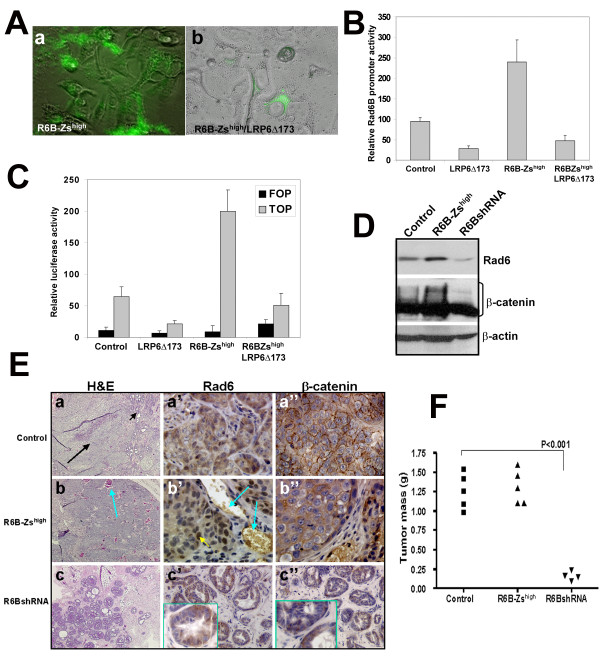
**Inhibition of Wnt signaling in WS-15 breast cancer cells selected for endogenous Rad6B overexpression downregulates Rad6B gene expression and β-catenin transcriptional activity**. (A) Fluorescence imaging of vector control R6B-Zs^high ^(a) or R6B-Zs^high^/LRP6D173 (b) WS-15 cells. (B) Relative Rad6B promoter activities in WS-15 subpopulations. (C) Relative pTOP/Flash or pFOP/Flash activities in WS-15 subpopulations. The data shown in C and D are averages (± S.E.M) of three separate experiments done in duplicate. (D) Steady-state levels of indicated proteins in the cytosols of vector control, R6B-Zs^high ^or PLKO-Rad6BshRNA WS-15 cells. (E) Tumor morphologies of vector control (a), R6B-Zs^high ^(b), and Rad6BshRNA (c) WS-15 xenografts by H&E staining. Immunohistochemical analysis of Rad6 (a', b' and c') and β-catenin (a", b" and c") in vector control, Rad6B-Zs^high ^and Rad6BshRNA WS-15 tumors. Short arrow in panel a shows hyperplastic region and long arrow shows invasive carcinoma. Long arrow in b and b' show blood vessels in tumors, and the short arrow in b' shows nuclear Rad6 staining. Note the loss of cell membrane staining of β-catenin in R6B-Zs^high ^tumors (compare a" and b"). Insets in c' and c" show magnified images of Rad6 and β-catenin staining, respectively, in hyperplastic ducts of Rad6BshRNA tumors. Original magnification ×4 (a and b); ×10 (c); ×20 (c' and c"); ×40 (a', a", b', b"). (F) Comparison of tumor masses of vector control, R6B-Zs^high ^and Rad6BshRNA WS-15 derivatives.

The functional link between Rad6B and Wnt/β-catenin signaling is not unique to MDA-MB-231 cells as indicated by a similar positive relationship in cells of the WS-15 human breast cancer line. Overexpression of mutant LRP6Δ173 in R6B-Zs^high ^WS-15 cells also induced a decline in ZsGreen-positive cells (Figure 5, compare Aa and Ab). Consistent with ZsGreen reporter expression, R6B-Zs^high ^WS-15 cells displayed ~2.4 fold higher Rad6B promoter-driven luciferase expression as compared to vector controls (*P *< 0.01), which was inhibited by ectopic expression of LRP6Δ173 (Figure 5B). TOP/Flash reporter assays showed high levels of endogenous β-catenin transcriptional activity in R6B-Zs^high ^WS-15 cells as compared to controls (*P *< 0.01), which was dramatically decreased by expression of mutant LRP6 (*P *< 0.01; Figure 5C). Western blot analysis showed the presence of higher steady state levels of Rad6 and high molecular weight β-catenin in R6B-Zs^high ^WS-15 cells as compared to vector controls, and a selective decrease in high molecular weight β-catenin fraction in Rad6B silenced WS-15 cells (Figure 5D). These data are consistent with our previous report that Rad6B silencing selectively decreases the polyubiquitinated high molecular weight forms of β-catenin without affecting the nascent 90 kDa band [22].

Control WS-15 cells implanted into the mammary fatpads of female nude mice produce large tumors within 60 days (Figure 5F). These tumors are composed of a small hyperplastic region (Figure 5Ea, short arrow) and a larger malignant area (Figure 5Ea, long arrow). Similar xenograft assays performed with R6B-Zs^high ^WS-15 cells produced large tumors (Figure 5F) that were generously populated with blood vessels (long arrows in Figure 5Eb and 5b'), and lacked the benign hyperplastic areas observed in control tumors (Figure 5E, compare a and b). Immunohistochemical analysis showed strong Rad6 staining in the cytoplasm of control tumors (Figure 5Ea') whereas Rad6 expression was observed in the cytoplasm and nuclei of R6B-Zs^high ^WS-15 tumors (short arrow in Figure 5Eb'). Control xenografts showed strong β-catenin staining in the cytoplasm and cell membranes (Figure 5Ea''), whereas R6B-Zs^high ^tumors showed strong cytoplasmic β-catenin expression with loss of staining on the cell membranes (Figure 5b''). WS-15-Rad6BshRNA cells produced significantly smaller tumors (*P *< 0.001) as compared to vector control or R6B-Zs^high ^WS-15 cells (Figure 5F). WS-15-Rad6BshRNA tumors were comprised of hyperplastic ducts and noticeably lacked the malignant region observed in vector control and R6B-Zs^high ^WS-15 tumors (compare Figure 5Ea and 5Eb with 5Ec). Rad6B silenced WS-15 tumors showed an overall decrease in Rad6 and β-catenin staining as compared to R6B-Zs^high ^and vector control WS-15 tumors (Figure 5Ec' and 5c'').

### Expression of Wnt regulated genes are influenced by Rad6B status

**Figure 6:**
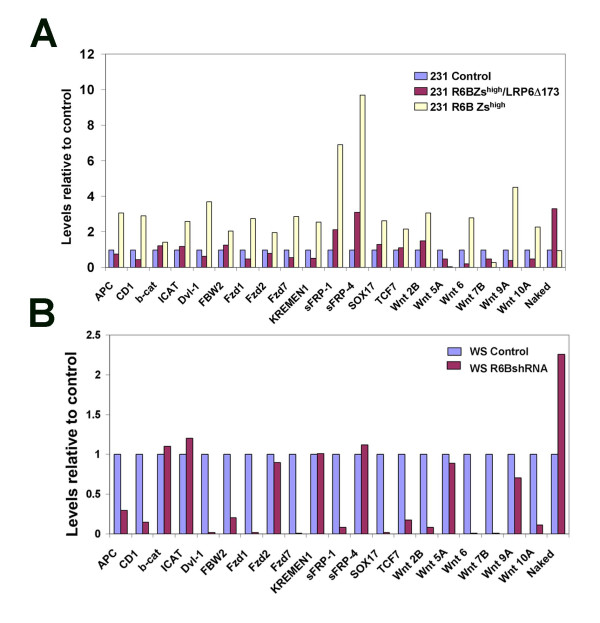
**Expression analysis of Wnt-regulated genes by RT^2 ^Profiler PCR SuperArrays**. (A) Relative levels in the indicated MDA-MB-231 (A) and WS-15 (B) derivatives.

To analyze the link between Rad6B and Wnt signaling pathway, we used RT^2 ^Profiler PCR SuperArrays to profile the expression of 84-Wnt related genes in vector control, R6B-Zs^high^, or R6B-Zs^high^/LRP6Δ173 MDA-MB-231 cells, or vector control or pLKO-Rad6BshRNA WS-15 [22] breast cancer cells. Expression of several Wnt signaling negative and positive regulators were increased in R6B-Zs^high ^MDA-MB-231 cells as compared to vector control, and disruption of LRP6 function in R6B-Zs^high ^MDA-MB-231 cells resulted in dramatic reduction in expression of these genes (Figure 6A). Wnt positive regulators Wnt 2B, Wnt 6, Wnt 9A, Wnt 10A, Fzd1, Fzd2, Fzd7, FBW2, Dvl-1, and cyclin D1 were upregulated in R6B-Zs^high ^MDA-MB-231 cells, and ectopic expression of mutant LRP6 in R6B-Zs^high ^cells resulted in a dramatic drop in expression of these transcripts (Figure 6A). Interestingly, expression of Wnt antagonists sFRP-1, sFRP-4, KREMEN1, and ICAT were elevated in R6B-Zs^high ^MDA-MB-231 cells and decreased in Wnt disrupted R6B-Zs^high ^MDA-MB-231 cells (Figure 6A). Similar analysis of Wnt signaling pathway regulators in empty vector control and pLKO-Rad6BshRNA WS-15 cells [22] validated the data from MDA-MB-231 cells. Expression of genes upregulated in R6B-Zs^high ^MDA-MB-231 cells, viz., Wnt 2B, Wnt 6, Wnt 10A, Dvl-1, Fzd1, Fzd7, sFRP-1, FBW2, SOX17, TCF7, and cyclin D1 were inhibited in Rad6B silenced WS-15 cells (Figure 6B). Expression of Naked, a negative regulator of Wnt signaling [29], was induced in WS-15/Rad6BshRNA cells and R6B-Zs^high^/LRP6Δ173 MDA-MB-231 cells relative to their corresponding controls (Figures 6A and 6B). β-catenin expression was unaffected by Rad6B overexpression (R6B-Zs^high ^MDA-MB-231 cells) or Rad6B silencing (Rad6BshRNA WS-15 cells), confirming our previous report that Rad6B induced β-catenin activation does not involve transcriptional events [22]. LRP5 and LRP6 expression was unaffected by Rad6B overexpression or silencing (data not shown). Similar changes in gene expression in MDA-MB-231 and WS-15 cells following Wnt disruption or Rad6B silencing, respectively, suggest that the expression of a set of genes that is regulated by β-catenin transcriptional activity is influenced by Rad6B status.

## Discussion

In this study, we provide novel evidence for a functional link between Wnt signaling and Rad6B in β-catenin activation, and the potential value of using Rad6B to target the Wnt/β-catenin pathway in human breast cancer. The data from this paper show that elevated β-catenin levels and activity observed in Rad6B-overexpressing breast cancer subpopulations (R6B-Zs^high^) require intact Wnt signaling since disruption of Wnt signaling in R6B-Zs^high ^cells with a signaling defective LRP6 lacking 173 amino acids in the c-tail [25] dramatically inhibits β-catenin levels and activity. The c-tail of LRP6 is a substrate for Wnt induced Dvl dependent phosphorylation [30-32]. The phosphorylated c-tail PPPSPXS motifs recruit Axin and GSK3β [31], and inhibits GSK3β by acting as competitive inhibitor of this kinase [33-36] with resultant accumulation of unphosphorylated β-catenin in the cytoplasm and translocation to the nucleus [4,37]. Rad6B is a transcriptional target of β-catenin [24]. Consistent with this, our present data show that inhibition of LRP6 signaling in R6B-Zs^high ^breast cancer cells induces a reduction in Rad6B promoter activity as evidenced by decrease in Rad6B promoter-mediated ZsGreen and luciferase reporter expressions and Rad6B gene expression.

Rad6B is overexpressed in human breast tumors [23,24]. To determine the functional contribution of Rad6B in breast cancer development and progression, in vivo assays were performed with MDA-MB-231 or WS-15 human breast cancer cells selected for high or low endogenous Rad6B expression. Although the tumors produced by R6B-Zs^high ^MDA-MB-231 cells are smaller than those from unselected control MDA-MB-231 cells, it is notable that R6B-Zs^high ^tumors are predominantly composed of spindle shaped cells that are typical of EMT, and capable of metastasizing spontaneously to the lung (1 out of 8) and lymph node (2 out of 8) of nude mice. Epithelial to mesenchymal transition plays a critical role in regulating cell migration during neoplastic invasion [38]. The Snail superfamily of zinc-finger transcription factors is essential for induction of EMT and invasive process [39]. Wnt signaling promotes tumor invasion by stabilizing Snail1 in breast cancer cells [40]. R6B-Zs^high ^MDA-MB-231 tumors show strong Snail1 expression, which is downregulated by mutant LRP6, whereas R6B-Zs^low ^MDA-MB-231 tumors lack the spindle shaped cells and express low or negligible Snail1. EMT is also frequently associated with loss of E-cadherin [41]. However, E-cadherin expression is very low in MDA-MB-231 cells [42]. Thus, it is likely that the epithelial mesenchymal transitions we have observed are a consequence of the nuclear activity of β-catenin rather than those involving interactions with E-cadherin.

The Wnt/β-catenin is frequently activated in human breast cancer. 40-60% of human breast cancers exhibit nuclear/cytoplasmic β-catenin and aberrant activation of the pathway at the receptor level is common [14,15,24,43-45]. LRP6 expression is upregulated in basal-like breast cancer [46]. Transgenic mice that overexpress LRP6 in mammary epithelial cells develop mammary gland hyperplasia, but fail to develop adenocarcinoma [47] suggesting the requirement for additional mechanisms for activating the Wnt pathway. Cooperation between Rad6B and intact Wnt signaling in breast cancer is apparent by the development of tumors with homogeneous EMT phenotype from R6B-Zs^high ^MDA-MB-231 cells as compared to R6B-Zs^high^/LRP6Δ173 derived tumors that are growth inhibited and lack the EMT phenotype. These findings are consistent with the data from Liu *et al *[20] that inhibition of LRP6 with the antagonist Mesd suppresses tumor growth and Wnt1 induced signaling. Our *in vivo *assays with WS-15 breast cancer cells further confirm the role of Rad6B in breast cancer progression since R6B-Zs^high ^WS-15 cells produced very angiogenic carcinomas as compared to controls, whereas Rad6B silencing inhibited progression to carcinoma with resultant benign hyperplastic tumors.

Our data from profiling of Wnt related genes show upregulation of several Wnt signaling pathway regulators in Rad6B overexpressing breast cancer cells, which are downregulated either by mutant LRP6 or Rad6B silencing. Wnt ligands are upregulated in subset of human breast cancers [20,48]. Since suppression of LRP6 in Rad6B overexpressing breast cancer cells results in decreases in expression of Wnt ligands (Wnt 2B, Wnt 6, Wnt 9A, Wnt 10A), β-catenin transcriptional activity and tumor growth suggests that overexpression of Rad6B and Wnt ligands may act in concert to influence breast cancer progression. Wnt5A expression was not affected by LRP6 suppression or Rad6B gene silencing, suggesting that Wnt 5A is not a critical regulator of the canonical Wnt pathway in MDA-MB-231 or WS-15 cells. The Wnt negative regulator sFRP-1 is reduced or lost in 80% of breast carcinomas [18,49]. However, our data showed that sFRP-1 is overexpressed in Rad6B overexpressing cells, and downregulated by Rad6B silencing or LRP6 suppression. This is consistent with a negative feedback response that may act to restrict the exposure of cells to a prolonged Wnt growth factor signal. Nevertheless, despite overexpression of sFRPs in R6B-Zs^high ^cells, the net outcome is one of increased β-catenin activity and tumor progression.

**Figure 7:**
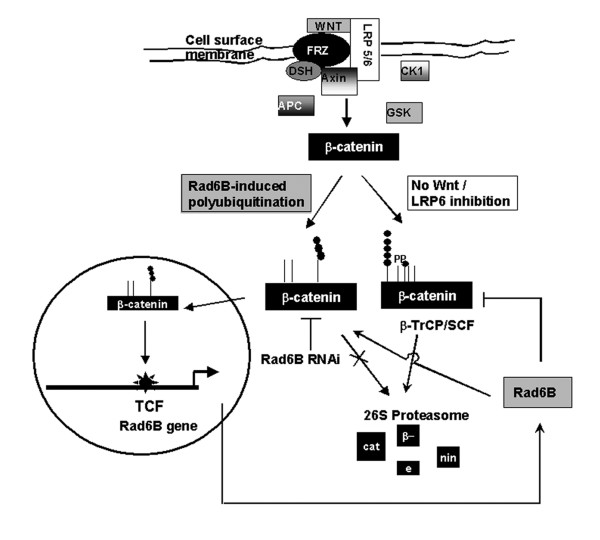
**Model of Rad6B/β-catenin regulation in breast cancer cells with autocrine Wnt activity**. Activation of the canonical Wnt pathway is regulated by Wnt ligand interactions with Wnt receptors Frizzled (FZD) and coreceptors LRP5/6 at the cell surface, and culminates in β-catenin stabilization by preventing its recruitment into the APC/Axin/GSK3 destruction complex. Rad6B transcription is induced by β-catenin, and Rad6B in turn stabilizes β-catenin by mediating K63 linked polyubiqitin modifications that render β-catenin insensitive to proteasomal degradation. Rad6B silencing disrupts the positive feedback loop between Rad6B expression and β-catenin stabilization with resultant decrease in ubiquitinated β-catenin and β-catenin transcriptional activity. The Rad6B induced β-catenin stabilization occurs downstream of Wnt activation and requires LRP6 mediated protection of β-catenin from incorporation into the destruction complex, as inhibition of LRP6 function in Rad6B overexpressors prevents β-catenin stabilization with resultant decreases in Rad6B expression.

The MDA-MB-231 cell line belongs to a subset of triple negative breast cancers that also express Vimentin [50]. This subset is thought to represent breast cancers that have undergone EMT and has been associated with clinical disease that is more invasive, has a higher mitotic index, and also a worse clinical outcome [51,52]. Rad6B gene expression is induced by TCF/β-catenin [24]. Rad6B in turn stabilizes β-catenin by polyubiquitin modifications that protect β-catenin from proteasomal degradation [22], thus setting off a vicious positive feedback loop between Rad6B expression and β-catenin activation (Figure 7). Our data demonstrate that selection of Rad6B overexpressing subpopulations selectively enriches for MDA-MB-231 subpopulations that exhibit homogeneous EMT phenotype and possess elevated β-catenin activity. Interference of Wnt signaling in Rad6B overexpressing subpopulations by a signaling deficient LRP6 decreases intracellular β-catenin, Rad6B gene expression, and inhibits EMT. Since similar outcomes on β-catenin activity and loss of EMT phenotype are achieved by Rad6B gene silencing [22], further confirm that Rad6B is a downstream mediator of β-catenin activation in the canonical Wnt signaling pathway (Figure 7).

## Conclusions

Taken together that similar outcomes on β-catenin activity, EMT suppression, or breast cancer progression are achieved by inhibiting LRP6 or Rad6B [22], illustrate the cooperation between the canonical Wnt signaling pathway and Rad6B in β-catenin activation. These data suggest that antagonizing Rad6B or LRP6 function may be beneficial for treatment of a subset of triple negative breast cancers or breast cancers with autocrine Wnt activity.

## Competing interests

The authors declare that they have no competing interests.

## Authors' contributions

BG participated in the generation of vector constructs and acquisition of data; PN participated in the in vivo assays; LT acquired, analyzed and interpreted immunohistochemistry and immunofluorescence data; MS conceived, designed and coordinated the study, acquired, analyzed and interpreted data, and drafted the manuscript. All authors read and approved the final manuscript.

## Grant Support

This work was funded by grants from the United States Department of Defense W81XWH07-1-0562 and W81XWH-09-1-0608 (MPS).
